# Percolation in Carbon Nanotube-Reinforced Polymers for Strain-Sensing Applications: Computational Investigation on Carbon Nanotube Distribution, Curvature, and Aggregation

**DOI:** 10.3390/ma16144959

**Published:** 2023-07-12

**Authors:** Alessandro Pontefisso, Michele Zappalorto

**Affiliations:** Department of Management and Engineering, University of Padova, Stradella San Nicola 3, 36100 Vicenza, Italy

**Keywords:** CNT, computational analysis, polymer composites, structural health monitoring

## Abstract

The present article investigates the possibility of simulating the electrical conductivity of carbon nanotube-reinforced polymer composites by numerical methods. Periodic representative volume elements are generated by randomly distributing perfectly conductive reinforcements in an insulating matrix and are used to assemble an electrical network representative of the nanocomposite, where the nanotube–nanotube contacts are considered equivalent resistors modeled by means of Simmons’ equation. A comparison of the results with experimental data from the literature supports the conclusion that a random distribution of reinforcements is not suitable for simulating this class of materials since percolation thresholds and conductivity trends are different, with experimental percolation taking place before the expectations. Including nanotube curvature does not solve the issue, since it hinders percolation even further. In agreement with experimental observations, the investigation suggests that a suitable approach requires the inclusion of aggregation during the volume element generation to reduce the volume fraction required to reach percolation. Some solutions available in the literature to generate properly representative volume elements are thus listed. Concerning strain sensing, the results suggest that representative volume elements generated with random distributions overestimate the strain sensitivity of the actual composites.

## 1. Introduction

The research for methods aimed at the Structural Health Monitoring, SHM, and strain sensing in polymeric composites is a bustling research field, where more than a hundred new articles are published every year. Considering a recent review of the damage monitoring methods available for fiber-reinforced polymer joints [1], among the several techniques investigated, the authors reported that the ones that are based on carbon nanotubes, CNTs, have attracted great interest in the last few years. These methods rely on the extraordinary mechanical and functional properties of CNTs and try to directly mix them with polymers to obtain materials with self-embedded sensors or try to create more intricate designs guiding their assembly at the nanoscale. In 2008, Böger et al. provided an early attempt at the former approach [2] by mixing an epoxy resin with carbon-based nanoreinforcements and using that compound for manufacturing glass fiber-reinforced epoxy laminates. Those laminates were electrically conductive and, by means of continuous monitoring of their conductivity during tensile and fatigue tests, the authors proved that it was possible to measure resistance changes directly related to microscale damage, such as inter-fiber failure. Nowadays, several research groups follow that approach, as proven by the many recent publications available involving various matrix materials, either polymeric or not, and different types of carbon-based reinforcements (e.g., [3,4,5,6,7,8,9]).

Polymer nanomodification by means of carbon nanotubes is a consolidated technique that results in extraordinary enhancements of the neat polymer mechanical and functional properties, as proven by decades of scientific research published on the topic [10]. There are, however, a few limitations that prevent the technique from becoming ubiquitous in the industry: CNTs are expensive to manufacture, they cause safety concerns [11], and they significantly increase the viscosity of the polymer matrix, negatively affecting its processability. The direct consequence of these issues is that CNTs are applied judiciously, limiting their quantity to the minimum amount possible. In addition, CNT-modified polymers usually exhibit a sudden transition in a property of interest (e.g., electrical conductivity) at a given CNT amount, and the scientific community dedicated significant efforts to developing methodologies for identifying that threshold condition for several material systems and target properties [12,13,14].

One of the most used research methods is based on the computational simulations of the nanocomposite material systems [12,15,16,17,18,19,20]. This method has a standardized structure, with a pre-processing phase in which the geometrical configuration of the material is embodied into a representative volume element, RVE, followed by a solution phase in which tools such as Finite Element Analysis are employed to generate data for a final post-processing phase, followed by the researcher deductions. With this structure, the ability of an RVE to be truly representative of a material system is of paramount importance for sustaining the validity of the results, and the choice of using a random distribution of particles, in place of other types of distributions, should be supported by strong evidence when generating the input for the solution phase. Nonetheless, in the literature, random distributions are commonly used by default, and discrepancies between the simulation results and experimental data are usually justified by unknown material parameters, or are adsorbed by best-fit procedures.

The literature about experimental data in CNT-reinforced polymers supports the idea that CNT distribution is neither homogeneous nor random. In his doctorate thesis dated 1998, Zhang [21] proposed the concept of dynamic percolation to refer to the dependency on time and temperature of the formation of conductive networks in polymer nanocomposites, a concept that he exploited in a series of research papers studying conductivity on several different thermoplastic polymers [22,23,24]. The underlying idea is that the polymer viscosity during the material processing plays a key role in determining the final composite conductivity, and this behavior is explained by CNTs’ mutual attraction. Other authors [25,26] reported how a CNT-modified polycarbonate exhibited a transition from insulator to conductor following a time-dependent reorganization of CNTs into the melted polymer. In [27], the authors studied the evolution of the conductivity in CNTs dispersed into silicone oil, providing more evidence of the tendency of CNTs to spontaneously create a conductive pathway. Martin et al. [28] investigated the formation of percolating networks in multi-wall CNT epoxy composites and provided evidence that electrical percolation appears at volume fractions significantly below those expected by the assumption of random distribution of particles. They explained the behavior in terms of local aggregation of CNTs, happening before the setting of the resin. Furthermore, they remarked that the processing parameters and the chemical nature of the composite constituents significantly affect the composite properties.

In the past few years, several researchers published articles in which they reported their findings about dynamic percolation from an experimental perspective [29,30], proving that the topic is of interest. However, from the computational perspective, the relevance of a proper characterization of the nanoparticle distribution is often overlooked, and the data presented in the literature are biased by algorithms that employ random patterns or do not offer a statistical description of the resulting RVE.

Considering the research on strain sensitivity of polymer/CNT composites through electrical measurements, several authors have contributed experimental and computational results. One of the earliest applications of these materials for macroscopic strain sensing was investigated by Dharap et al. [31], who provided evidence of a linear relation between stress and voltage in a composite film. Many other research activities were then published, investigating the effect of the material properties on strain sensitivity [20,32,33,34] and proving that this approach is of interest.

The present article investigates nanomodification-induced conductivity to compare the predictions based on RVEs generated with a random distribution of CNTs, with experimental data retrieved from the literature. At the same time, a way to infer the strain-sensing property of the nanocomposite is presented. Based on Simmons’ equation [35] to model the electrical conductivity of CNT contacts, the results support the idea that the random distribution is not suitable for representing these materials’ behavior, and that aggregation takes place regardless of the processing conditions employed. The direct consequence is that RVEs need to be constructed with particle distributions able to properly represent the material morphology in order to predict and investigate the material behavior. Eventually, some methods and computational tools available in the literature for generating RVEs with non-random distribution of reinforcements are briefly presented.

## 2. Theoretical Background

This section introduces the concepts of percolation, highlighting the difference between percolation from a geometrical perspective and percolation from a functional perspective. Then, the hypotheses and the procedure for constructing an electrical network representative of the RVE are presented alongside the physical equation used to infer the contact resistance between CNTs and a few parameters that will be used while discussing the analysis results.

### 2.1. Percolation in Nanocomposites

According to [36], the percolation threshold identifies the volume fraction at which CNTs develop a continuous network within the matrix, and it is of particular interest since it is usually coincident with the volume fraction at which the property enhancement due to nanomodification becomes appreciable. From a geometrical perspective, the concept of a continuous network underlies the need for contact between reinforcements. However, this constraint is not strictly mandatory for reaching electrical percolation, because it may not be required by the physical mechanism investigated: i.e., the electrical percolation is based on electrons tunneling through the insulating polymeric matrix and, as such, does not require physical contact between CNTs. It follows that to study the percolation condition, the knowledge of the mere interparticle distances is not enough, since the physical threshold distance for the electrical percolation in the material system being investigated plays a fundamental role.

To study percolation from a general perspective, this article distinguishes between a percolation distance related to the spatial distribution of reinforcements, d_GEOM_, and a percolation distance related to the physical mechanism of percolation in the material system, d_MAT_. The first one can be evaluated for any volume fraction, and it is defined as the minimum interparticle distance from which a percolative network can be geometrically identified across an RVE. The second one emerges from the addition of a physical property of interest (i.e., electrical conductivity), and it is related to the onset of the enhancement in the composite functional properties. To better clarify this difference, if assuming that a given RVE has an extremely low CNT volume fraction, it would not be conductive, but in this context, it would still be possible to identify d_GEOM_ for the given volume fraction: a continuous path from one face of the RVE to the opposite one can be identified moving through reinforcements at most X nanometers apart from each other, meaning that d_GEOM_ is X. Increasing the volume fraction, d_GEOM_ will decrease. At a given volume fraction, the electrical percolation is reached, and electrons can flow from one face of the RVE to the opposite one; d_GEOM_ reaches d_MAT_.

### 2.2. Modelling of the RVE Electrical Conductivity

The modeling of the material electrical conductivity in the following treatise is based on a few hypotheses:

1. The matrix is a perfect insulator, given the extremely low conductivity of an average polymer (e.g., 10^−8^ S/m [37]);
2. The electrical resistance of CNT contacts can be represented by a quantum mechanics phenomenon, where the current flows by means of the tunneling of electrons moving between two electrodes (i.e., the CNTs) separated by an insulator material (i.e., the matrix) [35];
3. CNTs are regarded as perfect conductors since their conductivity is orders of magnitude higher than that of a CNT contact (i.e., more than 10^4^ S/m [13]).

Under those hypotheses, the RVE can be regarded as an electrical network, where electrical joints among CNTs are considered electrical resistors, and the source and the sink of the electrical potential field are two opposite RVE faces (V_SOURCE_ and V_SINK_, respectively). The electrical resistance of the RVE is, therefore, the electrical resistance of the network. This system can be easily solved using Kirchhoff’s laws, as reported in other articles from the literature [13,38,39].

Worth mentioning is that the application of periodic boundary conditions requires some care: considering cubic RVEs, periodicity can be applied to faces that are not sink or source sites, which means four out of the six faces. Applying periodicity to the sink and source faces would cause an electrical shortcut. Therefore, three different networks for each RVE need to be constructed, one for each set of opposite faces.

### 2.3. Modelling of CNT Contact Resistance

To describe the electrical resistance of CNT contacts, Simmons’ equation is commonly used in the literature [13,39]. It can model the tunneling of electrons moving through a few nanometers of the insulator, such as in the case of CNTs dispersed in a polymer. The equation is

(1) $$R=\frac{h^{2}d}{ae^{2}\sqrt{2m∆}}\exp\left(\frac{4\pi }{h}d\sqrt{2m∆}\right),$$

where R is the electrical resistance of the contact, h is Plank’s constant, d is the interparticle distance, e is the charge of an electron and m is its mass, a is the contact area assumed equal to the square of the diameter of the nanotube, and ∆ is the energy barrier of the material system.

### 2.4. Parameters that Describe a CNTs RVE

To better understand the results reported in the Results Section, the parameters that characterize an RVE are defined here, and their mathematical relations are presented.

An RVE has a given CNT volume fraction, V_F_, defined as the ratio between the overall volume of the CNTs adsorbed in the RVE, Vol_CNT_, and the volume of the RVE itself, Vol_RVE_. Vol_CNT_ is calculated as the number of CNTs adsorbed in the RVE, N_CNT_, multiplied by the volume of one CNT, $L_{CNT}\pi D_{CNT}^{2}/4$, where a common constant length for the cylindrical CNTs, L_CNT_, and a common diameter, D_CNT_, are assumed. The aspect ratio, AR, of a CNT is L_CNT_/D_CNT_. From these considerations, it follows that

(2) $$V_{F}=\frac{N_{CNT}\;{Vol}_{CNT}}{Vol_{RVE}}=\frac{N_{CNT}\;\pi \frac{D_{CNT}^{2}}{4}L_{CNT}}{Vol_{RVE}}.$$

If CNTs are split into segments (a property that will be used in the next section), the equation still stands if L_CNT_ = N_SEGMENT_ ∙ L_SEGMENT_, with N_SEGMENT_ being the number of equal-length segments that divide the CNT, and L_SEGMENT_ their average length.

Since the RVE is a cube, Vol_RVE_ is calculated as the cube of its edge, Edge_RVE_. Moreover, it is possible to define a constant ratio between Edge_RVE_ and L_CNT_, β. Then, the following relations stand:

(3) $$V_{F}=\frac{N_{CNT}\;\pi \frac{D_{CNT}^{2}}{4}L_{CNT}}{{Edge}_{RVE}^{3}}=\frac{N_{CNT}\;\pi \frac{D_{CNT}^{2}}{4}L_{CNT}}{L_{CNT}^{3}\beta^{3}}=\frac{N_{CNT}\pi }{4AR^{2}\beta^{3}}.$$

Inverting the last equation, it is possible to obtain

(4) $$\frac{N_{CNT}}{\beta^{3}}=\frac{4}{\pi }V_{F}AR^{2}=N_{NORM}.$$

Since β depends only on the relative dimensions of CNT and RVE, it is possible to define a normalized number of reinforcements N_NORM_ as N_CNT_/β^3^, which allows us to describe the RVE only in terms of AR and V_F_. This last expression is used in the Results and Discussion Section.

## 3. Numerical Model

This section provides the numerical procedure for generating an RVE with random distributions of reinforcements, alongside the procedure for determining its geometrical percolation distance, d_GEOM_, and for assessing its electrical resistance. Eventually, an equation for determining the strain-sensing behavior of the composite is provided.

### 3.1. Generation of the Representative Volume Elements

The RVE generation is based on the ubiquitous Random Sequential Adsorption Algorithm, RSAA [40,41]. As the name suggests, it works by generating reinforcements with a random orientation and position within the RVE domain. The generation of reinforcements is sequential, which means that only one reinforcement is adsorbed into the RVE before a new one is generated. The reinforcement is checked for admissibility, verifying that no intersections with other reinforcements already adsorbed in the RVE are detected. If this condition stands, the reinforcement is adsorbed, and the RVE volume fraction increases accordingly. If intersections are detected, the reinforcement is discarded. This process is iterated until the target volume fraction is reached.

In more detail, in the present implementation of RSAA, CNTs are segmented into equal-length cylinders chained together. For modeling straight CNTs, each segment of the CNT is coaxial, while for modeling curved CNTs, each segment is tilted by a random solid angle within ±45 degrees with respect to the axis of the adjacent segment. It follows that the number of segments that divide the CNT affects the “magnitude” of its maximum global curvature.

**Figure 1:**
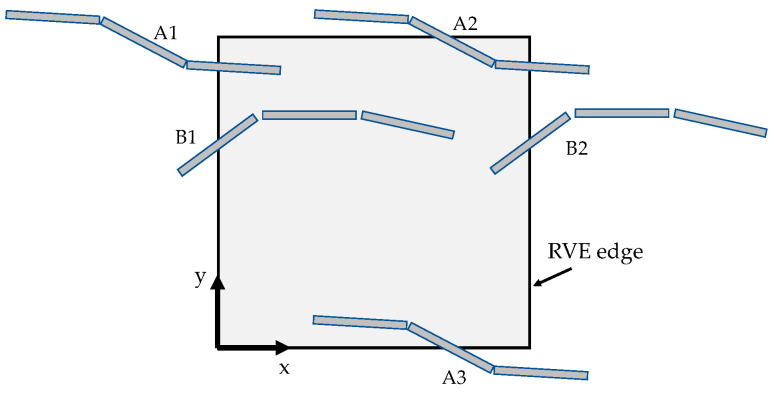
Representation of a bi-dimensional RVE with periodic instances. A1, A2, and A3 represent the same CNT, so that their combined area within the RVE is equal to one full CNT. The same concept stands for CNT B1 and B2.

Boundary conditions are periodic, meaning that CNTs can cross RVE faces, and in such events, periodic instances are generated in the RVE to preserve the expected CNT volume fraction (see Figure 1).

### 3.2. Calculation of the Interparticle Distances

The calculation of the interparticle distances relies on a geometrical routine that converts cylinders to polyhedrons, and then another routine that calculates the distance between such convex hulls [42]. This choice is based on the idea of developing a tool that can model complex shapes of arbitrary geometry, not just cylinders, a property that will be used in future research activities. For each new reinforcement, the distance from each of its segments to the segments of the reinforcements already adsorbed is recorded. Then, it is possible to find the minimum distance between each CNT to determine d_GEOM_. Relying on the property that the distance between two generic reinforcements is bijective, the calculation of interparticle distances can be performed incrementally, with a computational time that is O(N) for each iteration, with N number of reinforcement segments already adsorbed in the RVE (i.e., once a new reinforcement is adsorbed, the calculation of its distance from all the previously adsorbed reinforcements also provides the distance from each of the other reinforcements to the current one since the distance is the same).

### 3.3. Calculation of d_GEOM_ with the Graph Theory

The calculation of d_GEOM_ requires the search for the minimum interparticle distance from which a continuous network can be identified. Since each reinforcement may be in contact with every other reinforcement and RVE face, the determination of d_GEOM_ is not trivial: given N as the number of CNTs and RVE walls, there could be up to N!/2(N-2)! contacts. The solution that is implemented here is based on the graph theory [43,44], where the graph that represents the RVE is constituted by a set of nodes that contains all reinforcements and all RVE faces, and a set of edges that contains all distances between nodes. Edges are bijective relationships between nodes, and the search for a continuous pathway between V_SINK_ and V_SOURCE_ nodes can be made using a breadth-first search algorithm, BFS [44]:

1. After the selection of a test value for d_GEOM_, V_SOURCE_ is inspected to determine a list of nodes connected to it;
2. Then, the nodes of this list are further inspected to determine a new list of nodes connected to them to inspect;
3. This process is iterated until the next new list is an empty one, or it contains V_SINK_.

In the first case (i.e., the node containing V_SINK_ is not found), there is no continuous path between V_SINK_ and V_SOURCE_; conversely, in the second case, the path is discovered together with each reinforcement that is involved in its construction. To identify the minimum value for d_GEOM_, a search for a continuous pathway between V_SINK_ and V_SOURCE_ is performed running BFS with d_GEOM_ test values increasing from zero up to Edge_RVE_ until a percolation path is identified. For example, if there are two CNTs with an interparticle distance of 1.3 nm, they represent two nodes in a graph with an edge of length 1.3 nm that connects them. To find the minimum interparticle distance, it is possible to run the BFS algorithm with increasing tentative values of d_GEOM_ until it detects that at distances starting from 1.3 nm, the two nanotubes are connected. Extending this reasoning to RVEs of thousands of reinforcements is just a matter of computational effort. In [44], an open-source implementation of BFS is available for free, alongside examples to better understand the algorithm.

### 3.4. Calculation of the Electrical Conductivity

To determine the electrical conductivity of the RVE, the previously mentioned linear problem derived from Kirchhoff’s laws is solved. Since three networks exist for each RVE (one for each couple of opposite faces), three different values of electrical resistance can be determined. To aggregate the results, given the fact that a component will be constituted by a distribution of such a kind of RVE randomly oriented, the highest value for the resistance is regarded as representative of the simulated material. This choice is supported by the literature [45], and it implies that the material electrical resistance is determined by the highest value of d_GEOM_ among the three values calculated in the three periodic directions, d’_GEOM_.

Besides the calculation of the RVE electrical resistance by solving the electrical network by means of Kirchhoff’s laws, it is possible to approximate the resistance of the whole network with the electrical resistance obtained from a single Simmons’ equation which considers d’_GEOM_ as the value for the distance, taking advantage on the fact that the electrical resistance increases exponentially with the interparticle distance; thus, the electrical resistance of the junction at d’_GEOM_ is the highest one. This means that the overall RVE resistance may be approximated as the electrical resistance of the sole d’_GEOM_ junction since the other junctions in the network will give a smaller contribution. This solution is especially useful once a mathematical expression of d’_GEOM_ as a function of CNT volume fraction is known. Both this mathematical expression and a comparison between the two methods of calculating the RVE electrical resistance are reported in the next section.

Once the RVE electrical resistance is known, it is possible to evaluate the RVE electrical resistivity by dividing the resistance value by the distance between the sink and source faces and multiplying the result by the RVE cross-section. Given the fact that the RVE is a cube, its resistivity is obtained by multiplying its resistance by Edge_RVE_. Finally, the RVE conductivity can be obtained as the reciprocal of its resistivity.

### 3.5. Calculation of Strain-Sensing Effect in the VE

The calculation of the strain-sensing effect in the RVE is straightforward under the assumption that when there is an elastic deformation in the RVE, its volume changes, but its CNT volume fraction is the same. As such, it is possible to apply again the proposed model to infer the new electrical conductivity of the RVE along the loading direction, considering the new apparent volume fraction for CNTs.

Considering that the RVE initial volume is $V_{i}=l_{0}^{3}$ and the volume after elastic deformation is $V_{f}=l_{x}\cdot l_{y}\cdot l_{z}$, with $l_{0}$ being the edge length of the undeformed RVE and $l_{i}$ being the edge length after deformation in the i-th direction, it is possible to express V_f_ as

(5) $$V_{f}=V_{i}\cdot \left(1+\varepsilon_{y}\cdot \left(1-2\nu \right)+\varepsilon_{y}^{2}\cdot \left(\nu^{2}-2\nu \right)+\varepsilon_{y}^{3}\nu^{2}\right),$$

under the hypothesis of a deformation ε_y_ applied in the y direction.

## 4. Results and Discussion

### 4.1. Trend of Interparticle Distances in the Reference RVE

This subsection reports the findings of the research activity on the percolation condition of CNT-reinforced RVEs generated using the previously mentioned RSA algorithm. First, the case of straight CNTs is studied, followed by the case of curved CNTs. It will be shown that the introduction of curved CNTs has a detrimental effect on percolation—i.e., straight CNTs are more effective than curved CNTs in reaching the percolation condition.

**Figure 2:**
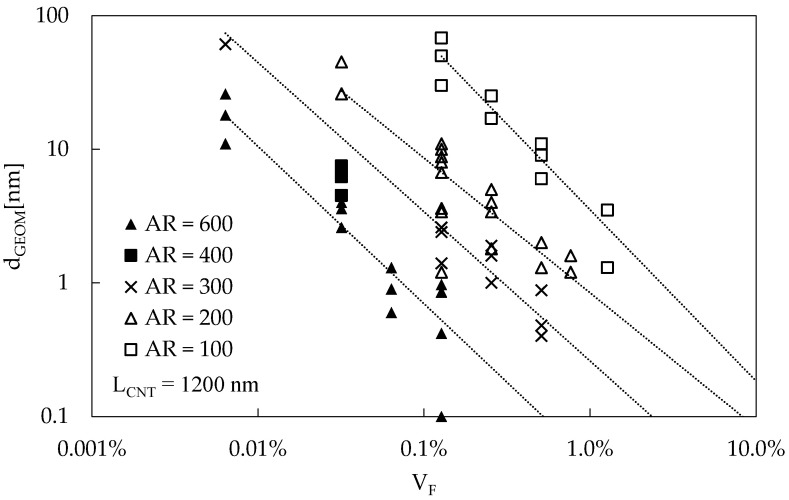
d_GEOM_ as a function of V_F_ for different values of the aspect ratio, AR. L_CNT_ = 1200 nm. Dotted lines represent regression lines of power law best fit applied to each value of AR.

Several RVEs were generated at different volume fractions and aspect ratios considering straight CNTs, following the procedure described in Section 3. The predicted values for d_GEOM_ of 22 simulations of RVEs are reported in Figure 2, where for each RVE, three points are plotted, representing d_GEOM_ calculated in the three periodic directions. The dotted lines represent power law regression curves for the average d_GEOM_ value of each RVE. In these simulations, the CNT length, L_CNT_, was 1200 nm, while the RVE edge was equal to 2400 nm. Such values were chosen following a convergence analysis, which proved to be coherent with that reported in [46].

**Figure 3:**
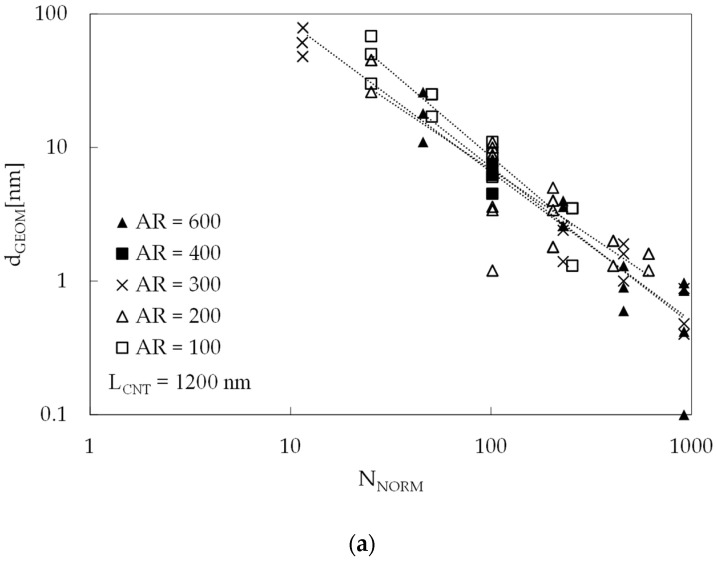

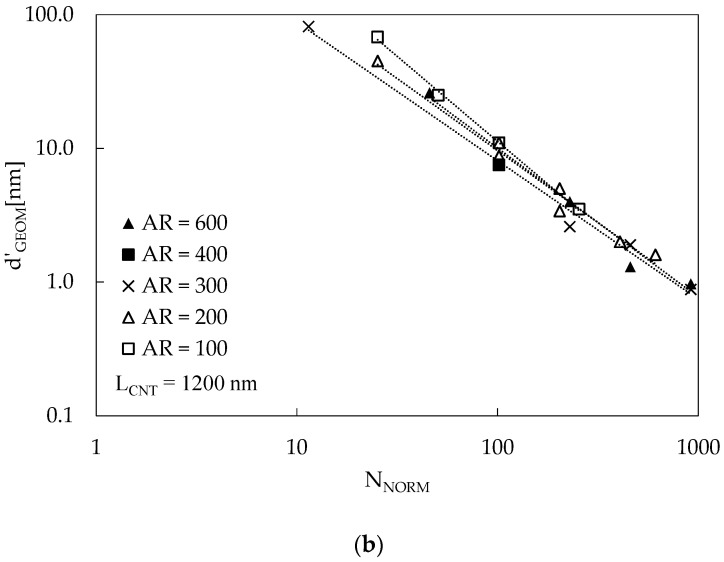
Same data as those reported in Figure 2, but with d_GEOM_ (**a**) and d’_GEOM_ (**b**) as a function of N_NORM_.

The trend of d_GEOM_ as a function of V_F_ is similar at all AR, as it is evident considering the dotted lines: increasing V_F_ and/or increasing AR, there is a reduction in the percolation distance d_GEOM_. Another interesting consideration emerges plotting d_GEOM_ as a function of N_NORM_, which is the parameter introduced in Equation (4), as in Figure 3a: the different values for d_GEOM_, as a function of AR and V_F_, are collapsed into a single band. This result is important since only one function is needed to describe the d_GEOM_ trend for all the simulated RVEs. The same results are reported in Figure 3b considering the sole d’_GEOM_, since it is the distance used in the electrical model. The trend is the same.

**Figure 4:**
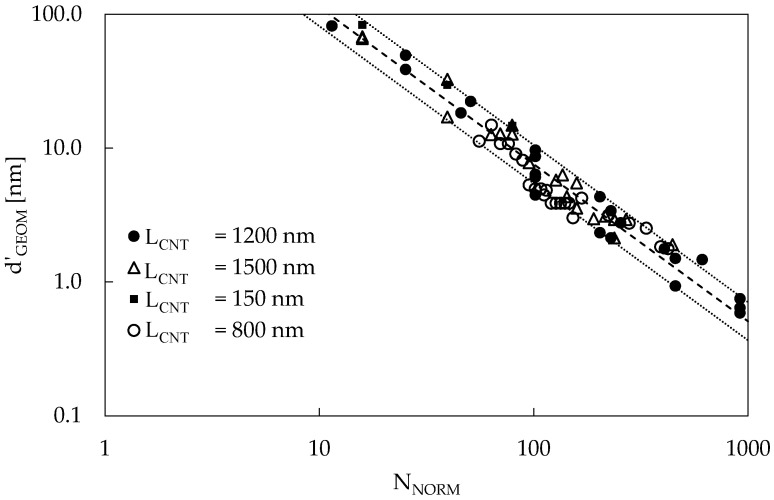
d’_GEOM_ as a function of N_NORM_ for different values of L_CNT_. d’_GEOM_ was scaled according to the factor L_CNT_/1200 nm. Three series for different AR values are plotted for each L_CNT_ value. The dashed line represents the power law best fit of the average values, while the dotted lines represent 10% and 90% probability.

To assess the validity of this band, even accounting for an L_CNT_ change, new RVEs were generated considering reasonable values for CNT length (such as 150, 800, 1200, and 1500 nm). The results are reported in Figure 4 in terms of d’_GEOM_ as a function of N_NORM_. The d’_GEOM_ for each different value of L_CNT_ was conveniently scaled up (or down) with a multiplying factor equal to L_CNT_/1200 nm to show that, using such scaling, the new data points belong to the same band. Under this condition, the existence of the proposed band appears to be independent of L_CNT_; thus, it is possible to use it to infer the interparticle distance for every combination of L_CNT_, AR, and V_F_.

**Figure 5:**
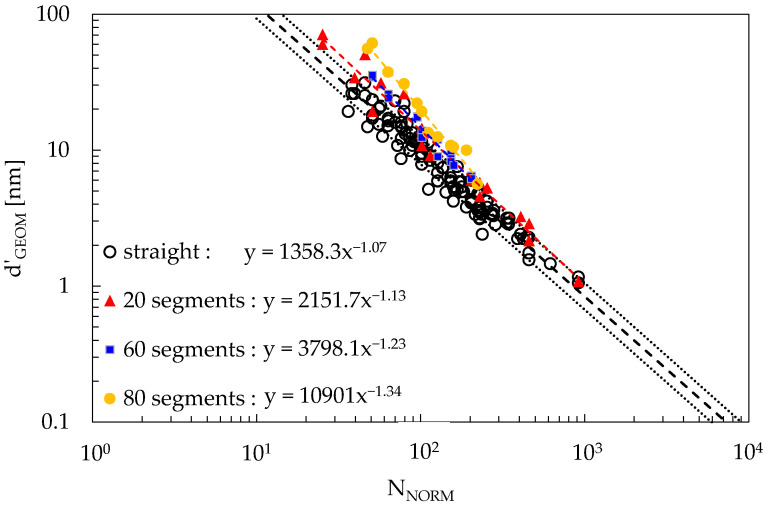
Plots for d’_GEOM_ as a function of N_NORM_ for different values of CNT curvature, as reported in the legend. The case of straight segments aggregates all data reported in the previous figures, while for the case of curved CNTs, three RVEs were generated for each curvature, with AR = 100, 200, and 600, and Edge_RVE_ = 2400 nm. The dashed line represents the power law best fit of the average values, while the dotted lines represent 10% and 90% probability.

Regression curves based on power laws fitted to d’_GEOM_ in simulated RVEs are reported in Figure 5, where the results for the case of straight and curved CNTs are reported.

Considering that values for d_MAT_ reported in the literature [39] in the case of electrical percolation are above 1 nm [35], it is revealed that straight CNTs perform better than curved CNTs, since a lower V_F_ is needed to reach electrical percolation at a given AR value. This result agrees with other results available in the literature [45,47] that show how including CNT curvature hinders electrical percolation, thus supporting the soundness of the approach being used.

### 4.2. Calculation of Electrical Conductivity in a Case Study

The idea that the simulated values of the electrical resistance for an RVE can be calculated either by solving the electrical network using Kirchhoff’s laws or applying Simmons’ equation directly to d’_GEOM_ was previously discussed in Section 3.4: d’_GEOM_ is associated with the highest resistance in the network, and it is unlikely that the overall resistance is much higher than that since Equation (1) predicts an exponential trend. If this approach is validated, the reduction in computational cost would be significant.

**Figure 6:**
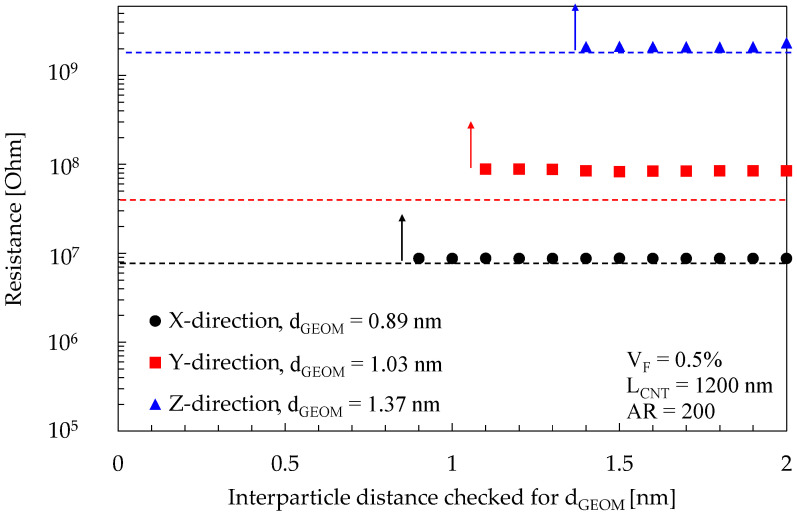
Points represent the simulated value for the RVE resistance as a function of d_GEOM_, obtained by solving the electrical network with Kirchhoff’s laws in the three RVE directions, while the dashed lines represent the electrical resistance value as obtained from a single Simmons’ equation that considers as distance just d_GEOM_ of the given direction.

To support the validity of the assumption, Figure 6 shows a comparison of the predicted values for the RVE resistance as obtained by the two approaches. The figure reports the case of an RVE with V_F_ = 0.5%, L_CNT_ = 1200 nm, AR = 200, and energy barrier 1 eV. Three datasets are reported in the image, considering the same RVE simulated in the three periodic directions, alongside the estimation of the electrical resistance of the sole junction at d_GEOM_ according to Equation (1). At an interparticle distance greater or equal to d_GEOM_, the electrical resistance assumes a finite value since the BFS algorithm finds a path connecting the source and sink faces. Such resistance is kept practically constant even if additional contacts may be added to the network at distances greater than d_GEOM_. Adding those contacts means, from an electrical perspective, that new resistors are being placed in a parallel configuration with respect to the existing ones, but since they have a significantly higher electrical resistance, they would only marginally affect the overall resistance of the RVE.

The maximum error in the estimation of the electrical resistance is detected for the y direction, where the resistance estimated with Equation (1) is of about 3E7 Ohm, while the resistance directly evaluated from solving the electrical network is of about 9E7 Ohm. Upon further examination, the discrepancy is due to the presence of other junctions of length close to that of d_GEOM_ in a series configuration. To account also for this condition, a possible approach should consider the statistical distribution of junction lengths that characterize the particle distribution and apply a correction factor. Eventually, in the following part of the manuscript, the material conductivity values were evaluated directly by applying Simmons’ equation to d’_GEOM_.

**Figure 7:**
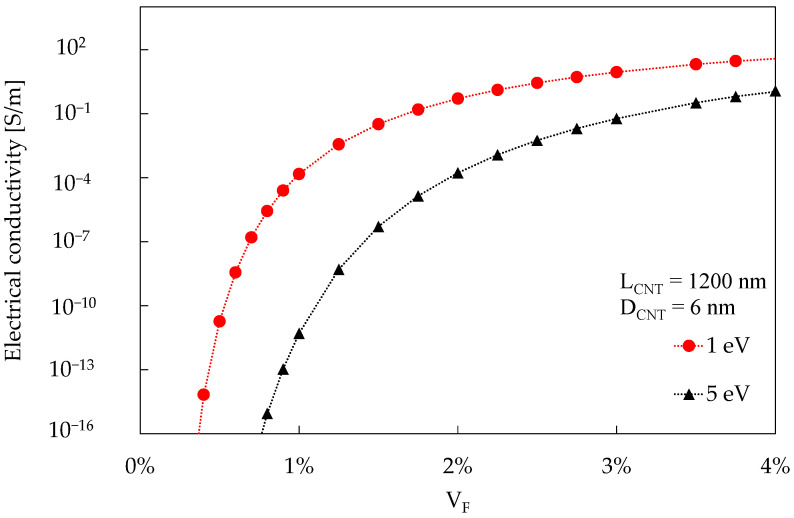
Electrical conductivity prediction obtained using a hard-core random process for generating the CNT RVE and Simmons’ equation. Two values for the energy barrier are reported: 1 eV and 5 eV.

With the proposed procedure, it is then possible to estimate the electrical conductivity for a CNT-reinforced polymer as a function of V_F_ by just providing a value for AR, D_CNT_, and Simmons’ equation energy barrier. An example is reported in Figure 7, where the electrical conductivity of a polymer containing CNTs of length 1200 nm and diameter 6 nm is simulated for two values of energy barrier, namely 1 eV and 5 eV, as a function of the CNT volume fraction.

As far as the value of the energy barrier is concerned, it results in a curve translation, preserving the overall trend. Thus, since its values depend on the material system being considered, and since the literature reports values generally up to 5 eV [16,39,48], a value of 1 eV will be used in Section 4.4 of the paper, deeming that this choice does not hamper the following considerations about the CNT distribution.

### 4.3. Strain-Sensing Effect

**Figure 8:**
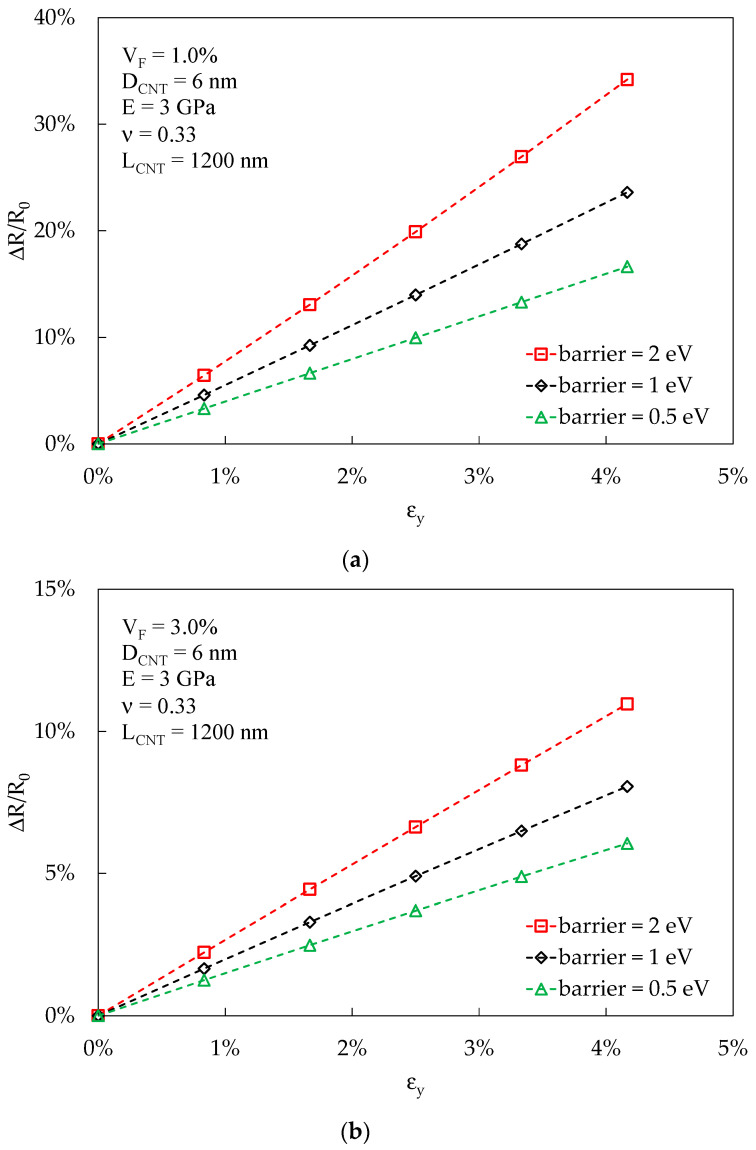
Variation of electrical resistance in an RVE subjected to tension in y direction, as predicted by the proposed approach. Elastic, morphological, and electrical properties are reported on the images. In (**a**), a volume fraction of 1% is considered, while in (**b**), it is 3%.

The proposed methodology is useful for estimating the effectiveness of a composite blend in detecting the strain applied to the material. Following the approach proposed in Section 3.5, the strain-sensing effect based on the specimen’s electrical conductivity can be studied very straightforwardly. An example is reported in Figure 8a,b, in the case of an RVE subjected to tension in the y direction, with the x and z directions free to deform according to Poisson law. ε_y_ represents the strain applied to the RVE. Elastic properties are representative of an average epoxy resin, with the Young modulus E = 3 GPa, and Poisson coefficient ν = 0.33, whereas the reinforcements are CNTs of length 1200 nm and diameter 6 nm at two different volume fractions (1% in Figure 8a and 3% in Figure 8b). Three different values for the energy barriers are reported: 0.5 eV, 1 eV, and 2 eV. The model predicts that a higher energy barrier, at volume fractions above percolation, results in a higher strain-sensing sensitivity, defined as the ratio between the RVE electrical resistance variation, ΔR, and the RVE initial electrical resistance, R_0_. At the same time, a higher V_F_ negatively affects the sensitivity to strain. These results are congruent with the trends exhibited in Figure 7, where a higher slope of the curves corresponds to a higher sensitivity to strain. These results are coherent with those reported in [48], where the authors presented analytical and experimental evidence of the described trends, and the results reported in [39], where the increase in the height of barrier potential led to a more sensitive piezoresistivity. Considering reference [33], where a computational investigation on strain sensitivity in CNT-modified polymers is compared with experimental data, the increase in CNT V_F_ again resulted in a decrease in strain sensitivity, and the overall magnitude of strain sensitivity agrees with that predicted by the proposed methodology.

### 4.4. Comparison with Experimental Results

Besides the methodology proposed for estimating the strain-sensing capability of a CNT-reinforced polymer, this manuscript’s goal is to investigate the effect of the CNT distribution and curvature on the conductivity of the polymer. In the previous sections, it was presented how the curvature in CNTs is detrimental to reaching electrical percolation. However, one aspect that is still undiscussed is if a random distribution, such as that obtained by RSA algorithms, is properly representing these kinds of composites. This subsection attempts to shed light on this aspect using comparisons with experimental data retrieved in the literature.

**Figure 9:**
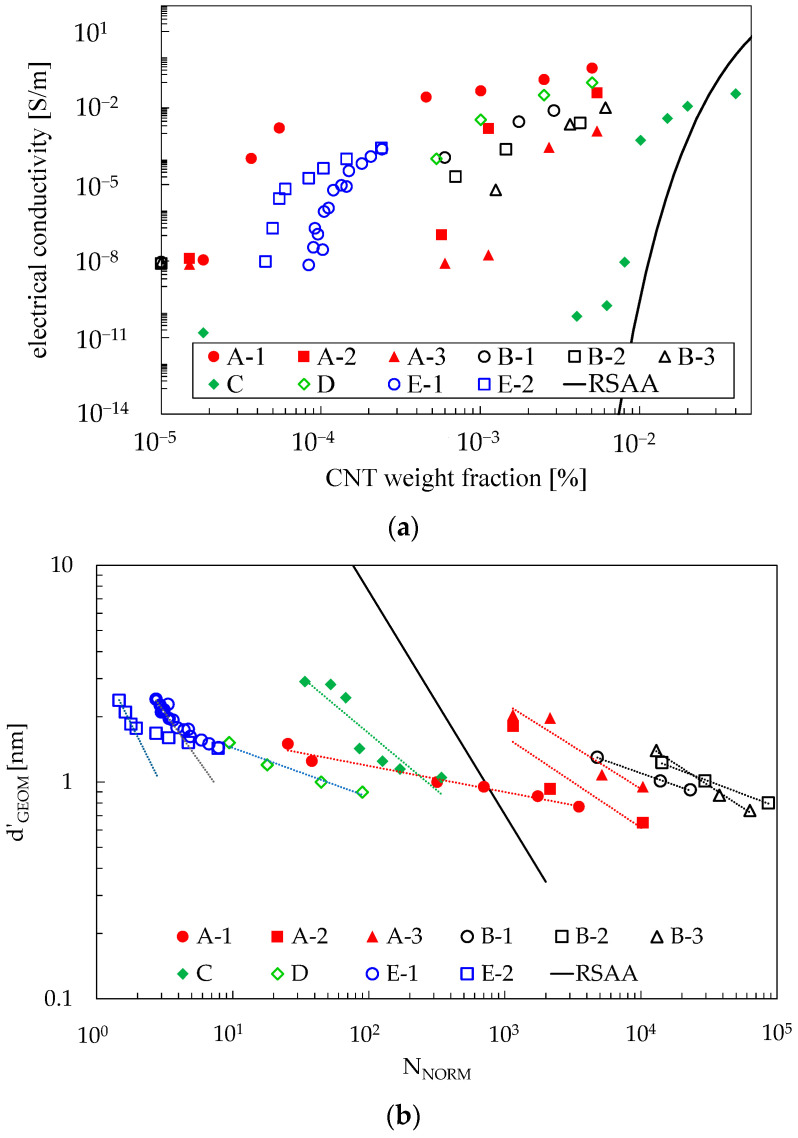
Values of the electrical conductivity (**a**) and inferred values for d’_GEOM_ (**b**) obtained from several experimental data according to the proposed methodology. For the correspondent references see Table 1.

The presented approach for simulating the material conductivity from d’_GEOM_ may be reversed: once the experimental conductivity of a CNT-reinforced polymer is known, it is possible to obtain a value for d’_GEOM_, inverting Simmons’ equation. To this end, a value for the energy barrier needs to be assumed, and for comparing heterogeneous data from the literature, a constant value of 1 eV was used. Under those hypotheses, the results presented in the previous section can be compared with results from the literature once V_F_, L_CNT_, and D_CNT_ are known. The comparison is reported in Figure 9 and Table 1, where data about conductivity were taken from [27,37,48,49,50] for single-walled, multi-walled, and dual-walled CNTs obtained by different researchers with different manufacturing processes. To convert the CNT volume fraction to weight fraction, a density of 1.2 g/cm^3^ was assumed for the polymer, and 2.2 g/cm^3^ was assumed for CNTs. In all cases but [27], the matrix is Epoxy resin, which is a polymer suitable for structural applications thanks to its relatively high mechanical properties and which would benefit from the strain-sensing property obtained by nanomodification. Conversely, in [27], the matrix is a silicone oil employed for the precise purpose of studying dynamic percolation. In addition, [27]-1 and [27]-2 represent the same composite tested 17 and 700 s after stirring, respectively. These measurements are meant to prove that the variation of the polymer viscosity with time is of paramount importance when processing CNT-reinforced polymers, since differences in electrical properties may be due to a morphological reconfiguration of the reinforcements.

Figure 9a provides an overview of the composites’ conductivity considering CNT weight fractions, but such a representation is not able to take into account the different lengths and aspect ratios of CNTs. Figure 9b elaborates on the data of Figure 9a, inferring d’_GEOM_ as a function of N_NORM_ to filter out the effects of different CNT lengths and aspect ratios, following the considerations of Section 4.1. Considering that the interparticle distance at which electrical percolation is reached in this class of materials (i.e., d_MAT_) is between 1 and 2 nm (as reported in [39]), it can be seen that such a condition is reached regardless of the actual N_NORM_. This outcome is supported by the experimental results and conclusions reported in [28], where the authors observed that CNTs spontaneously form aggregates that subsequently agglomerate, resulting in a macroscopic network covering large volume fractions of the epoxy. Moreover, the authors remarked that long CNTs and short CNTs follow different mechanisms. It means that the real behavior of these materials cannot be properly represented by an RVE generated with random distributions of reinforcements.

**Figure 10:**
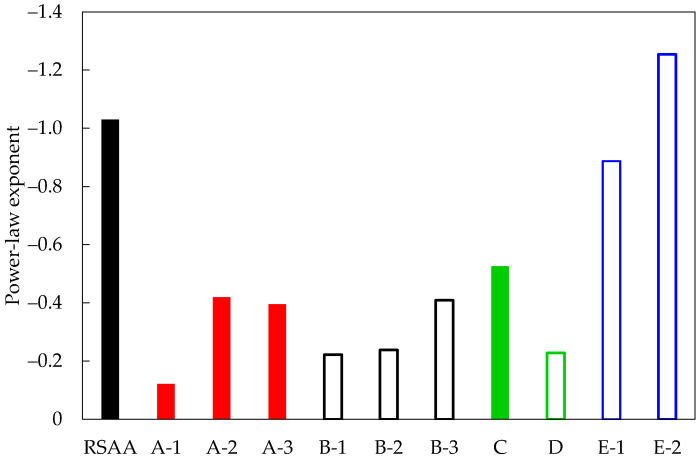
Histogram of the power law exponents reported in Table 1.

To bring further evidence to the limitations of the RSA algorithm in modeling CNT-modified polymers, in Figure 10, a comparison regarding the slope of the best-fit curves plotted in Figure 9b is presented. Two facts are clear:

1. Data obtained by various sources show a consistent trend, with a slope exponent higher than −0.5 regardless of the processing, the material, and the research group which published the results. The only results appreciably below that value are those reported in [17], where the authors focused explicitly on dynamic percolation and only considered data at extra-low volume fractions, where CNT interactions are reduced (in fact, the best-fitting curves plotted in Figure 9b account only for the lowest volume fractions);
2. The model created by RSAA is not representative of the experimental data obtained by commonly used processing conditions, according to its power law exponent.

To better describe the effect of the exponent, it should be considered that increasing N_NORM_ in a composite material (e.g., increasing the volume fraction of CNTs) has a lower effect on its conductivity if the exponent tends to zero. This is usually of little importance since percolation is reached nonetheless, but it affects the capabilities of the material to have a high sensitivity to strain, as previously discussed. The slopes of d’_GEOM_ for experimental data can be explained if considering that CNTs promote clustering when dispersed into a polymeric matrix. This clustering has a positive effect at low V_F_ since it promotes percolation. On the other hand, the same clustering hinders the ability of CNTs to homogeneously occupy the space within the matrix, and the increase in V_F_ is less effective than it could be without CNTs’ mutual attraction. The CNTs aggregation is also the cause of the fact that in the literature, the experimental trend of conductivity results in an asymptotic value at increasing amounts of CNTs after percolation. These considerations find support in papers such as [28,37], where the authors investigated the formation of percolating networks in multi-wall CNT epoxy composites and provided evidence that electrical percolation appears at volume fractions significantly below those expected by the assumption of random distribution of particles. They explained the behavior in terms of local aggregation of CNTs, happening before the setting of the resin. Furthermore, they remarked that the processing parameters and the chemical nature of the composite constituents significantly affect the composite properties.

An interesting consideration is that the trend of d’_GEOM_ could not be explained by the presence of CNTs’ curvature since that would increase the slope of the curves with respect to the case of straight CNTs, as was discussed in Section 4.1. As far as the y intercept is concerned, the different values exhibited by experimental data find an explanation both in terms of the different processing parameters, which affect the final AR of the CNTs (e.g., by breaking CNTs, and thus resulting in L_CNT_ values different from the nominal ones) and in the different materials, which affect the correct value for the energy barrier in Simmons’ equation. However, neither effect can result in a variation in the curve slope: as proved in Section 4.1, the CNT aspect ratio does not affect the slope of the simulated curve as much as the value of the energy barrier, which is a material constant superimposed to the d’_GEOM_(N_NORM_) curve, cannot modify it.

Since the trend of the electrical conductivity of the material as a function of V_F_ is directly correlated to its strain sensitivity, it follows that the actual distribution of CNTs unavoidably affects the real performance of a composite. Eventually, the results support the idea that an RVE obtained with the RSA algorithm has a higher electrical sensitivity to strain as compared with real composites since, past percolation, the electrical conductivity of the real composites is less sensitive to a variation in volume fraction, and thus to deformation. This result is a direct consequence of the slope of the experimental curves: while dynamic percolation bolsters a material’s electrical conductivity, the resulting electrical network is less affected by deformations.

**Figure 11:**
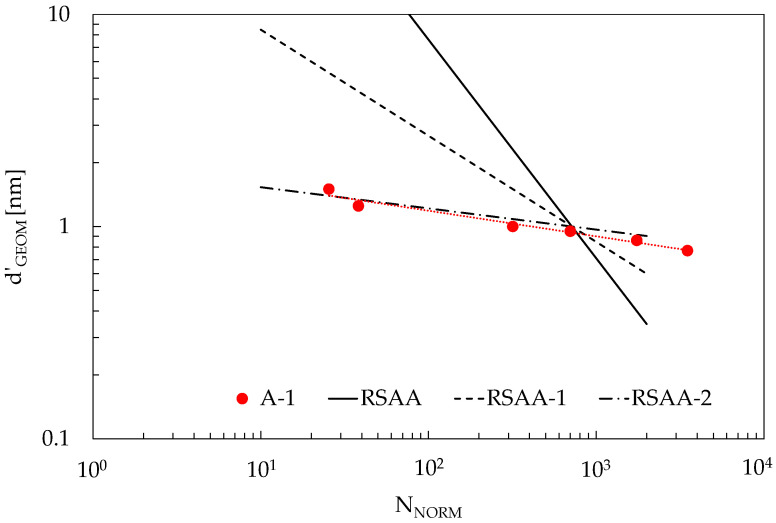
Values for d’_GEOM_ obtained from different power laws, together with the trend of A-1 already reported in Figure 9b.

To clarify these trends and provide a quantitative assessment of the effect of the slope coefficient on the sensitivity to strain, Figure 11 presents three different power laws characterized by different slope coefficients but sharing the same d’_GEOM_ at an assumed percolation threshold of 1 nm (=d_MAT_). For comparison, the figure reports the trend of [49]-1 already reported in Figure 9b to highlight the similarity with an experimental condition. In Table 2, the values for the power law coefficients and exponents of each curve are reported. The same table reports a value for the sensitivity to strain, defined as the average slope of the ΔR/R_0_(ε_y_) function evaluated as in Section 4.3 for a strain from zero up to 4%, for two different volume fractions (1% and 3%), L_CNT_ = 1200 nm, AR = 200, E = 3 GPa, ν = 0.33, and barrier 1 eV.

The results show that, as expected, increasing the CNT volume fraction beyond percolation conditions results in a material with a progressively reduced sensitivity to strain. Assuming that the references reported in Table 1 are representative enough of common applications, meaning that experimental values for the power law exponent in the range of [−0.1, −0.5] cover a broad range of applications, RVEs constructed with random distributions of reinforcements may overestimate strain sensitivity from about 2 to 10 times.

An interesting perspective would be to try to hinder dynamic percolation during the material processing to gain a higher strain sensitivity.

### 4.5. Final Remarks and Possible Solutions

A final consideration is that to effectively simulate this kind of nanocomposite using computational simulation, randomly distributing CNTs in RVEs is not representative of the actual distribution of reinforcements. Efforts should be dedicated to the identification of the realistic morphology of CNTs and their computational reproduction using a suitable control over CNT placement. As far as the identification of the morphology is concerned, electron microscopy may be used to collect representative images of the material. Since these images are usually bi-dimensional, a Generative Adversarial Network can be used to reconstruct a realistic 3D RVE [51]. Another possible approach is presented in [52], where the authors propose an approach that relies on molecular dynamics to generate a realistic distribution of CNTs without relying on morphological information acquired by experimental techniques. A third possible solution may be proposed on the basis of statistical considerations derived from microscopy observations. It consists of the generation of RVEs with enhanced RSA algorithms obtained by modifying the algorithm reported in [41] for the case of spherical nanoparticles, which is based on the modification of the resulting distribution of reinforcements using an additional aggregation step in-between reinforcement adsorptions. In terms of implementation, it works in the same way as the RSA algorithm, randomly distributing reinforcements in the matrix in sequential order, but for each particle that is placed into the VE, the algorithm searches for other particles that were previously placed near this last one, which is moved as if under the effects of a gravitational attractive force. The magnitude of the aggregation force and the corresponding movement can be controlled by comparing the current distribution with the target distribution by functions such as those reported in [40], where statistical descriptors are used for nanoplatelet distribution and can be readily adapted to the case on nanotubes.

## 5. Conclusions

In the present manuscript, RVEs of CNT-reinforced polymers were generated according to the random distribution of nanotubes in cubic domains. The interparticle distance that allows geometrical percolation within an RVE was determined by means of the graph theory as a function of CNT lengths, aspect ratios, and volume fractions, and it was expressed by a single power law fitting against a single parameter, the normalized number of reinforcements. Applying Simmons’ equation, the electrical conductivity of the RVEs was determined and, inverting the procedure, the percolation distances of experimental datasets were inferred. Comparing the geometrical percolation distance of the proposed random distribution of straight and curved CNTs against the experimental ones, it was found that neither straight nor curved CNTs explain the trend in experimental data. A different explanation was proposed based on CNTs’ dynamic percolation, with nanotubes dispersed into polymers exhibiting aggregation and developing interparticle distances significantly below those expected by random distribution at low-volume fractions. Leveraging on a proposed model for strain-sensing estimation, it is expected that RVEs generated with a random distribution of CNTs overestimate the strain-sensing performance of a composite from about 2 to 10 times. A final consideration is that the ubiquitous Random Sequential Adsorption Algorithm is not suitable by itself for generating representative morphologies for this kind of composite, since it fails to account for the attractive forces that promote the generation of a continuous network of CNTs, thus predicting electrical percolation at volume fractions higher than those shown by experimental testing, as much as predicting a higher strain sensitivity. To overcome this limitation, tools able to enforce and control the aggregation of reinforcements into RVEs need to be used in order to generate volume elements that are truly representative of the material under investigation.

## Figures and Tables

**Table 1:** The third and the fourth columns report the coefficient, C, and the exponent, E, of the best-fitting power law equation d’_GEOM_ = C(N_NORM_)^E applied to the experimental data of [27,37,48,49,50] and plotted in Figure 9b. Material column indicates the kind of CNT; L_CNT_ and D_CNT_ columns list the value reported in the referenced articles. Processing synthetically describes the material processing used in the referenced publication. The first row reports the results of the computational method discussed in this article.

Label	Reference	Power Law Coefficient	Power Law Exponent	Material	L_CNT_ [μm]	D_CNT_ [nm]	Processing
RSAA	RSAA	873	−1.03	-	1.2	6	RSA Algorithm
A-1	[49]-1	2.07	−0.121	MWCNT	50	50	high-speed shear mixing
A-2	[49]-2	29.6	−0.42	SWCNT	3	2	sonication in ethanol + high-speed shear mixing
A-3	[49]-3	35.2	−0.395	SWCNT	3	2	ball milling + high-speed shear mixing
B-1	[37]-1	8.49	−0.222	MWCNT	37.5	15	3-roll milling
B-2	[37]-2	11.8	−0.238	SWCNT	8	2	3-roll milling
B-3	[37]-3	66.8	−0.409	DWCNT	8	2.8	3-roll milling
C	[50]	18.9	−0.526	MWCNT	1.4	13	magnetic stirring + sonication
D	[48]	2.4299	−0.228	MWCNT	1.5	9.5	high-speed shear mixing + sonication
E-1	[27]-1	5.815	−0.887	MWCNT	1.5	9.5	sonication + magnetic stirring
E-2	[27]-2	3.857	−1.254	MWCNT	1.5	9.5	sonication + magnetic stirring

**Table 2:** Power law exponents and coefficients for the curves reported in Figure 11, together with the results of a strain-sensing sensitivity analysis at different CNT volume fractions.

Curve	Power Law Exponent	Power Law Coefficient	V_F_	Strain Sensitivity
RSAA	−1.03	873	1%	5.7
RSAA-1	−0.5	26.8	1%	2.2
RSAA-2	−0.1	1.93	1%	0.37
RSAA	−1.03	873	3%	1.9
RSAA-1	−0.5	26.8	3%	1.3
RSAA-2	−0.1	1.93	3%	0.33

## Data Availability

The data that support the findings of this study are available on request from the corresponding author.
